# Recurrent Brain Metastasis Versus Radiation-Induced Necrosis: A Case Report and Literature Review

**DOI:** 10.7759/cureus.34400

**Published:** 2023-01-30

**Authors:** Hisham Sweidan, Abdullah Jarrah, Aron Weingarten, Feng Zhu, Sarah AlQasem, Nouraldeen Manasrah, Ahmed Jamal Chaudhary

**Affiliations:** 1 Internal medicine, Detroit Medical Center/Sinai Grace Hospital/Wayne State University, Detroit, USA; 2 Internal Medicine, Detroit Medical Center/Sinai Grace Hospital/Wayne State University, Detroit, USA; 3 Medical School, Wayne State University School of Medicine, Detroit, USA; 4 Internal Medicine, Luzmila hospital, Amman, JOR; 5 Internal Medicine, Detroit Medical Center/Sinai Grace Hospital, Detroit, USA

**Keywords:** upper extremity weakness, aphasia, metastatic non-small cell lung cancer, brain metastasis, brain radiation necrosis

## Abstract

Radiotherapy is the cornerstone of brain metastasis management. With the advancement of therapies, patients are living longer, exposing them to the long-term effects of radiotherapy. Using concurrent or sequential chemotherapy, targeted agents, and immune checkpoint inhibitors may increase the incidence and severity of radiation-induced toxicity. Recurrent metastasis and radiation necrosis (RN) appear indistinguishable on neuroimaging, making it a diagnostic dilemma for clinicians. Here, we present a case of RN in a 65-year-old male patient who previously had brain metastasis (BM) from primary lung cancer, misdiagnosed initially as recurrent BM.

## Introduction

Radiation necrosis is increasingly recognized and should be kept in mind, particularly when the patient has symptoms resembling recurrence [1]. With the advancement of therapies, patients are living longer, exposing them to the long-term effects of radiotherapy. Some studies suggest increased frequency with the use of biological agents and immune checkpoint inhibitors [2]. Treatment-induced necrosis represents a serious side-effect. Symptoms are produced by localized brain necrosis and reactive edema. Differentiating recurrent tumors from tissue necrosis can be very difficult by imaging, and both entities are primarily indistinguishable on conventional structural MRI and have similar clinical presentations [3]. Radiation-induced changes can appear up to 10 years following initial treatment [4]. Here, we illustrate how the case can be elusive and difficult to distinguish from recurrent metastasis. 

## Case presentation

**Figure 1:**
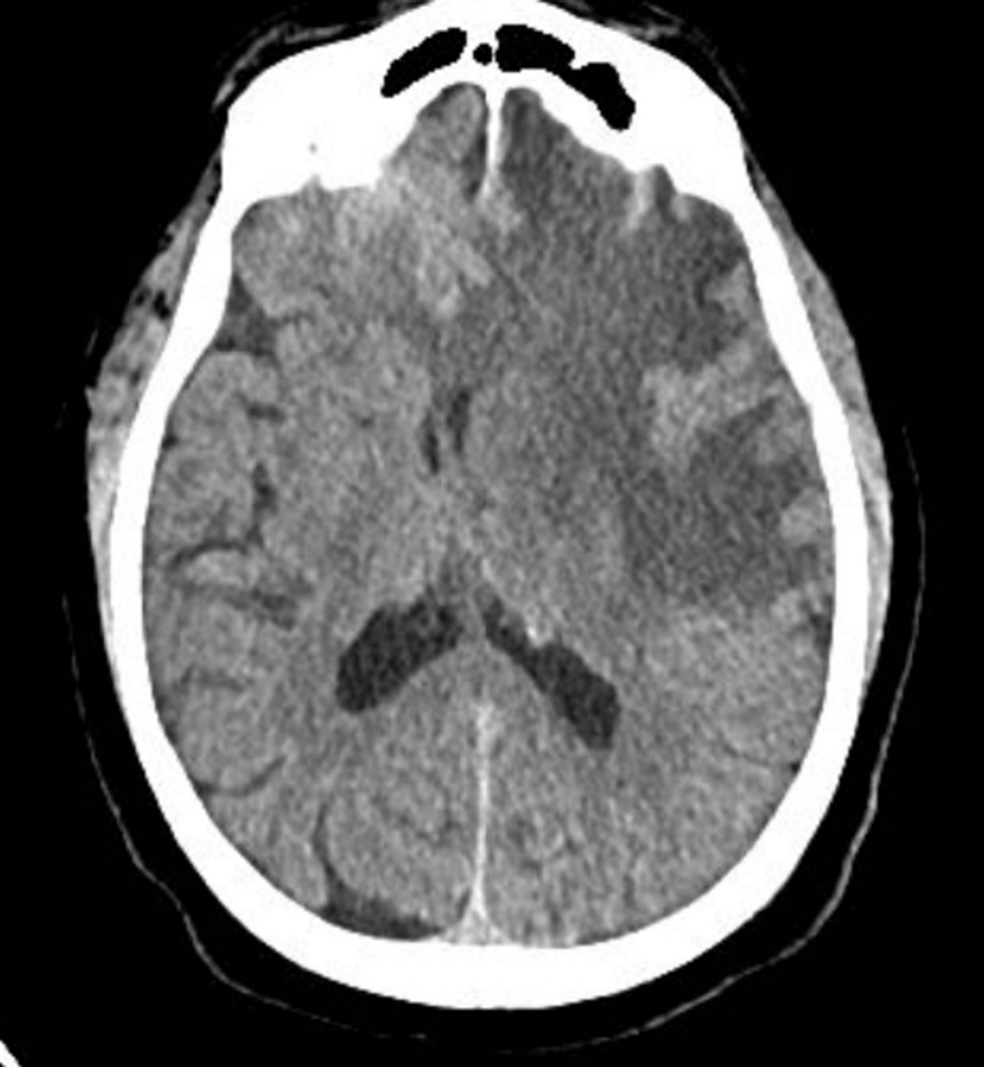
Head CT was done on presentation, showing hypo attenuated area and mass effect

A 67-year-old male presented with a five-day history of right-sided upper and lower extremity weakness, aphasia, and headache. He had a history of non-small cell lung cancer with brain metastasis which was diagnosed in 2020. He was initially treated with chemotherapy, followed by radiation. Radiation was completed in January 2021 with concurrent carboplatin and paclitaxel. Unfortunately, he developed recurrent stage IV disease with brain metastasis. When he presented to the emergency department, he was on pembrolizumab. His vitals were within normal limits. Physical examination revealed 0/5 right upper and lower extremity motor strength with intact sensation and cranial nerve function. The rest of the physical exams were normal. Complete blood count and basic metabolic panel were within normal ranges. Head CT showed a large region of hypoattenuation centered within the left frontal lobe with a 9 mm midline shift and effacement of the anterior horns of the lateral ventricles (Figure 1).

**Figure 2:**
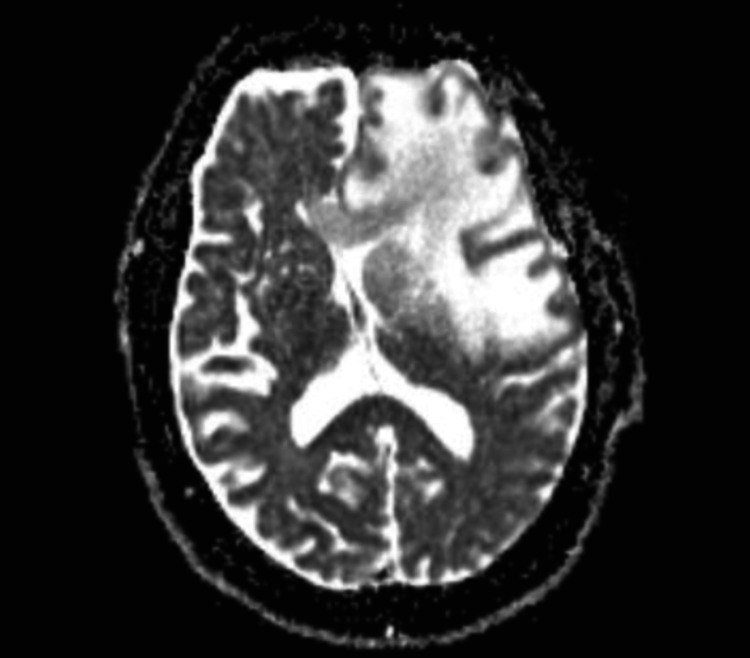
Brain MRI shows peripherally enhancing lesion noted anteriorly on the left extending to the cortical surface with new significant surrounding vasogenic edema

Head MRI revealed a peripherally enhancing lesion anteriorly on the left extending to the cortical surface with new significant surrounding vasogenic edema (Figure 2). The findings were thought to represent recurrent metastatic disease with extensive vasogenic edema. 

**Figure 3:**
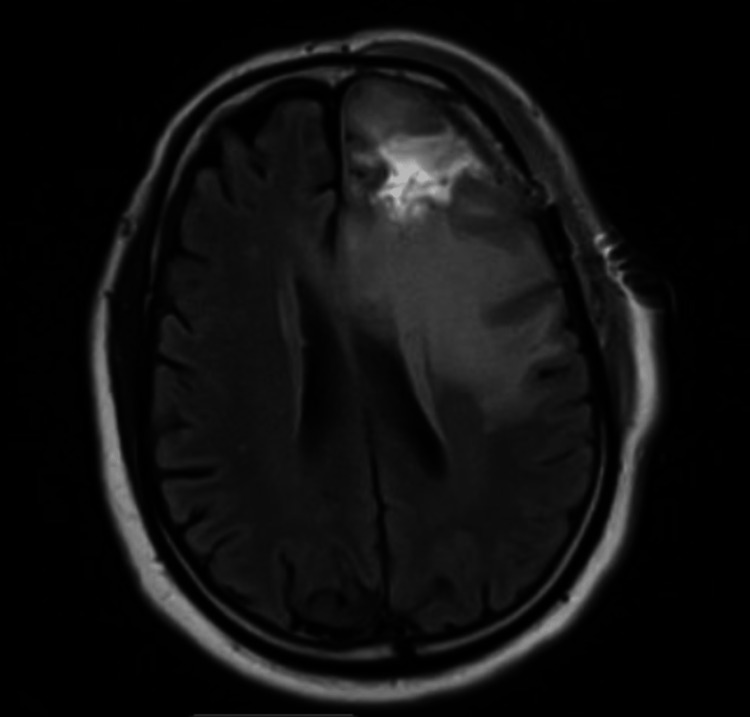
Post-operative MRIs show diminished mass effect

The patient was stabilized for transfer to the general medical floor. High-dose steroids and Levetiracetam were started to prepare the patient for surgery. The patient underwent left frontal stereotactic craniotomy with gross total resection of the brain lesion and computer-assisted navigational procedure for resection of the intradural brain mass. No complications were associated with surgery. He continued to show marked recovery, regained most of the strength of the right side, and improved his speech. Post-tumor resection MRI status was significant for a resection cavity anteriorly on the left, with no definite residual enhancement (Figure 3).

**Figure 4:**
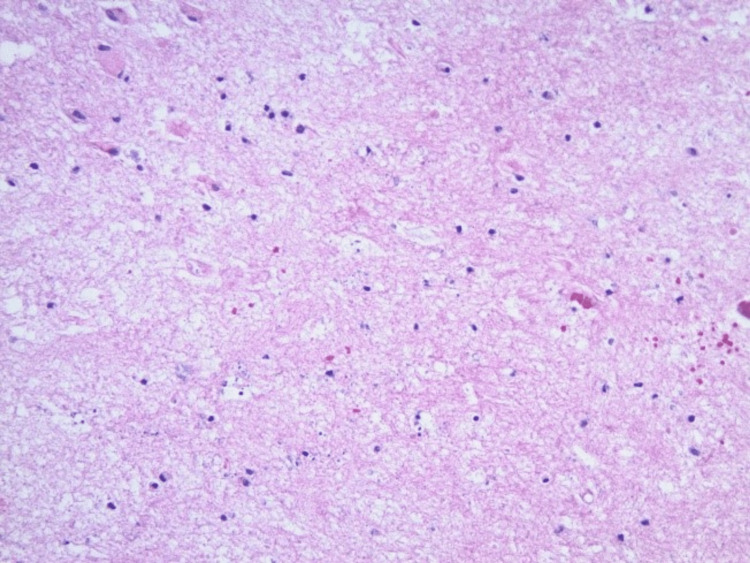
Post-treatment changes with no malignant cells to suggest metastasis.

Overall, the diminished mass effect was significant. A final pathological examination from specimens obtained during surgery revealed no features suggesting tumor recurrence (Figure 4).

The patient’s symptoms significantly improved, such that he ambulates well without assistance, and his speech has returned to baseline. He was enrolled in inpatient rehabilitation before discharge. Upon discharge, outpatient occupational and physical therapy were arranged. Follow-ups with oncology and neurosurgery, as well as 3-month postoperative brain MR imaging, were scheduled, and the patient was discharged with tapering doses of steroids and Levetiracetam.

## Discussion

Radiation necrosis is a complication of radiotherapy that can resemble tumor recurrence. Delayed RN can be seen several months to years after initiating radiotherapy up to 10 years following therapy. Risk factors depend on radiation dose, and the volume irradiated [5]. For example, lesions below ≤ 20 mm can be safely treated with 24 Gy, 21-30 mm with 18 Gy, and 31-40 mm with 15 Gy. However, a dose of more than 55Gy has serious side effects and a higher risk to develop radiation necrosis [6]. Tissue necrosis is due to the consequence of vascular endothelial cell damage, resulting in fibrinoid necrosis of small vessels and direct brain parenchymal necrosis. These conditions appear within the irradiated volume as contrast-enhancing, expansive brain lesions surrounded by edema [7].

The differential diagnosis for brain-enhancing lesions includes but is not limited to recurrent brain metastasis, primary brain tumor, or infectious process (abscess). Another differential is intracerebral hemorrhage, which was excluded based on initial CT findings. Infection with abscess formation was excluded as the patient was afebrile, with a normal white blood cell count, and with negative meningeal signs on physical examination. Radiation-induced necrosis confirmed by pathology excluding recurrent brain metastasis or primary brain tumors as possible differential diagnosis. Symptoms produced by localized brain necrosis depend upon the location of the lesion. They can include focal neurologic deficits, progressive motor weakness, speech disturbance, or more generalized symptoms like headache, nausea, and increased intracranial pressure. Seizures are a common presentation in up to 20% of patients [8].

MR perfusion, MR spectroscopy, or positron emission tomography (PET) can be used for diagnosis [9]. Ultimately, a biopsy of the suspicious lesion may be required for a definitive diagnosis, particularly in patients who are symptomatic and have worsening imaging findings over time [10]. In some cases, tissue necrosis is an asymptomatic, self-limited process. Initial treatment with a moderate dose of glucocorticoid is recommended in symptomatic patients. Follow-up imaging in one to two months is generally recommended. Various other treatment options like bevacizumab and laser interstitial thermal therapy (LITT) are viable treatment options that can be utilized [11].

Our patient was presented with clinical features suspicious of recurrent brain metastasis, prompting a thorough workup. Given the patient’s history, clinical presentation, and imaging suggestive of cerebral edema with a midline shift, surgical resection was deemed necessary and the optimal intervention. Pathology revealed no evidence of malignancy and was consistent with post-radiation changes. Clinicians must be aware of post-radiation necrosis. Survival of patients with BM is being prolonged with improved systemic therapy and increased use of stereotactic radiosurgery (SRS), clinically manifesting with late effects of radiotherapy.

## Conclusions

Radiation therapy is increasingly being utilized for patients with metastatic disease. Unfortunately, it can damage normal tissues in rare cases leading to radiation necrosis. Differentiating tumor growth from radiation necrosis can be difficult but is essential to determine to provide appropriate patient management. Corticosteroids, surgery, and bevacizumab are all possible effective treatment options. Clinicians should choose the treatment that offers the patient resolution of neurologic systems with minor toxicity and invasiveness. 
