# Correction: Recurrent Brain Metastasis Versus Radiation-Induced Necrosis: A Case Report and Literature Review

**DOI:** 10.7759/cureus.c123

**Published:** 2023-06-15

**Authors:** Hisham Sweidan, Abdullah Jarrah, Aron Weingarten, Feng Zhu, Sarah AlQasem, Nouraldeen Manasrah, Ahmed Jamal Chaudhary

**Affiliations:** 1 Internal medicine, Detroit Medical Center/Sinai Grace Hospital/Wayne State University, Detroit, USA; 2 Internal Medicine, Detroit Medical Center/Sinai Grace Hospital/Wayne State University, Detroit, USA; 3 Medical School, Wayne State University School of Medicine, Detroit, USA; 4 Internal Medicine, Luzmila hospital, Amman, JOR; 5 Internal Medicine, Detroit Medical Center/Sinai Grace Hospital, Detroit, USA

This article has been corrected at the request of the authors to include Aron Weingarten as third author. Mr. Weingarten was erroneously omitted from the author list upon initial submission and publication. The authors apologize for this error.

